# Monitoring of pulmonary involvement in critically ill COVID-19 patients - should lung ultrasound be preferred over CT?

**DOI:** 10.1186/s13089-022-00299-x

**Published:** 2023-02-26

**Authors:** Arthur W. E. Lieveld, Micah L. A. Heldeweg, Jasper Schouwenburg, Lars Veldhuis, Mark E. Haaksma, Rutger M. van Haaften, Berend P. Teunissen, Jasper M. Smit, Jos Twisk, Leo Heunks, Prabath W. B. Nanayakkara, Pieter Roel Tuinman

**Affiliations:** 1 Section Acute Medicine, Department of Internal Medicine, Amsterdam UMC, Location VU Medical Center, Postbox 7507, 1007MB Amsterdam, The Netherlands; 2 Department of Intensive Care Medicine, Amsterdam University Medical Centers, Location VU Medical Center, Amsterdam, The Netherlands; 3 Section Emergency Radiology, Department of Radiology and Nuclear Medicine, Amsterdam UMC, Location VU Medical Center, Amsterdam, The Netherlands; 4 Department of Epidemiology and Data Science, Amsterdam UMC, Location VU Medical Center, Amsterdam, The Netherlands; 5 Amsterdam Leiden IC Focused Echography (ALIFE), Amsterdam, The Netherlands

**Keywords:** COVID-19, Point-of-cafe ultrasound, Lung ultrasound, Computed tomography, Monitoring, Mortality

## Abstract

**Background:**

It is unclear if relevant changes in pulmonary involvement in critically ill COVID-19 patients can be reliably detected by the CT severity score (CTSS) and lung ultrasound score (LUSS), or if these changes have prognostic implications. In addition, it has been argued that adding pleural abnormalities to the LUSS could improve its prognostic value. The objective of this study was to compare LUSS and CTSS for the monitoring of COVID-19 pulmonary involvement through: first, establishing the correlation of LUSS (± pleural abnormalities) and CTSS throughout admission; second, assessing agreement and measurement error between raters for LUSS, pleural abnormalities, and CTSS; third, evaluating the association of the LUSS (± pleural abnormalities) and CTSS with mortality at different timepoints.

**Methods:**

This is a prospective, observational study, conducted during the second COVID-19 wave at the AmsterdamUMC, location VUmc. Adult COVID-19 ICU patients were prospectively included when a CT or a 12-zone LUS was performed at admission or at weekly intervals according to local protocol. Patients were followed 90 days or until death. We calculated the: (1) Correlation of the LUSS (± pleural abnormalities) and CTSS throughout admission with mixed models; (2) Intra-class correlation coefficients (ICCs) and smallest detectable changes (SDCs) between raters; (3) Association between the LUSS (± pleural abnormalities) and CTSS with mixed models.

**Results:**

82 consecutive patients were included. Correlation between LUSS and CTSS was 0.45 (95% CI 0.31–0.59). ICCs for LUSS, pleural abnormalities, and CTSS were 0.88 (95% CI 0.73–0.95), 0.94 (95% CI 0.90–0.96), and 0.84 (95% CI 0.65–0.93), with SDCs of 4.8, 1.4, and 3.9. The LUSS was associated with mortality in week 2, with a score difference between patients who survived or died greater than its SDC. Addition of pleural abnormalities was not beneficial. The CTSS was associated with mortality only in week 1, but with a score difference less than its SDC.

**Conclusions:**

LUSS correlated with CTSS throughout ICU admission but performed similar or better at agreement between raters and mortality prognostication. Given the benefits of LUS over CT, it should be preferred as initial monitoring tool.

**Supplementary Information:**

The online version contains supplementary material available at 10.1186/s13089-022-00299-x.

## Background

Management of critically ill patients with Coronavirus Disease 2019 (COVID-19) requires accurate and appropriate clinical care while maintaining adequate infection control and minimizing patient harm. Chest computed tomography (CT) is the gold standard imaging modality to diagnose and monitor COVID-19 pneumonia [1, 2]. However, CT requires patient transport to and from radiology. This carries considerable risks for critically ill patients, and healthcare workers, while laying claim to already stretched resources and personnel [3, 4]. In contrast, point-of-care ultrasound (POCUS) offers the unique possibility to perform imaging studies at the bedside, obviating the need for transport. POCUS also has disadvantages: in untrained hands there is risk of interpretative error and the equipment may be microbially contaminated if not conscientiously used [5].

Quantification of pulmonary involvement by lung ultrasound (LUS) has shown to be equivalent to CT [6, 7]. Baseline lung ultrasound score (LUSS) is associated with ICU admission, hospital and ICU length of stay, ARDS and even mortality (when included in a composite end-point) [6–10].

However, it is neither clear if clinically relevant changes in the degree of pulmonary involvement over time can be reliably detected by the LUSS and CT severity score (CTSS), nor if these changes have prognostic implications for mortality in critically ill COVID-19 patients. Literature on monitoring of the disease course by CT and LUS, is limited to mostly small and retrospective case series, with a short follow-up time [11–16]. In addition, there are hardly any studies that investigate the reliability and measurement error of the CTSS and LUSS [6, 11, 16]. Furthermore, it has been argued that pleural line abnormalities and subpleural consolidations should be incorporated into the LUSS, since they are often found in patients with COVID-19 and correlate histologically with diffuse alveolar damage, and a such might have prognostic value [10, 16–18].

Our aim was to fill these knowledge gaps by comparing LUSS and CTSS for the monitoring of the pulmonary involvement of critically ill COVID-19 patients during their admission through: first, establishing the correlation of LUSS (± pleural abnormalities) and CTSS to monitor pulmonary involvement throughout admission; second assessing agreement and measurement error between raters for LUSS, pleural abnormalities, and CTSS; third, evaluating the association of the LUSS (± pleural abnormalities) and CTSS with mortality at different timepoints during ICU admission.

## Methods

### Study design and setting

This is a prospective, observational study conducted at the academic ICU of the Amsterdam UMC, location VUmc between October 20, 2020 and February 20, 2021. The study was approved by the Medical Ethical Committee of the VUmc, along with a waiver of informed consent (2020.011). The trial was registered in the Dutch trial registry (NL8540).

### Patients

Consecutive adult patients (≥ 18 years) with a laboratory confirmed diagnosis of COVID-19 were eligible for inclusion upon admission to ICU. Patients were included and followed when a chest CT or LUS were performed at admission or at one of the weekly intervals afterward. Patients were excluded when: COVID-19 was not the primary reason of admission (incidental finding), no CT or LUS was performed, or ≤ 6 lung zones were scanned. Start of follow-up was the first LUS or CT, whichever came first. Patients were followed up for 90 days by chart review or until death.

Patient characteristics along with ventilator settings and laboratory values were collected from the electronic patient database at baseline and at the time of the CT. Please see Additional file 1 for our COVID-19 treatment protocol.

### Chest computed tomography (CT)

It was standard practice for patients to receive a chest CT every week when their clinical condition did not improve or deteriorated. The CT was made to determine the amount of pulmonary involvement, and assess the presence of a possible super-infection or pulmonary embolism. CTs were evaluated by local radiologists with varying degrees of experience and access to clinical information—but not to the LUS examinations. A visual assessment of the percentage of pulmonary involvement in each lobe was summed for a total CT severity score (CTSS), used in the internationally validated COVID-19 Reporting and Data System (CO-RADS), ranging from 0 (no involvement) to 25 (maximum involvement), as described previously [1]. If a super-infection was suspected on CT, a trained and experienced pulmonologist would perform a broncho-alveolar lavage (BAL) at the location of the suspected super-infection. The decision to perform a CT (or a subsequent BAL) was made by the treating ICU consultant in consultation with a daily multi-disciplinary team consisting of a consultant microbiologist, a consultant pulmonologist and at least five other ICU consultants. Please see the Additional file 1 for the CT scan protocol.

### Lung ultrasound (LUS)

LUS is a part of standard care on our ICU [19]. The examination was performed within 24 h of the CT to minimize temporal influence on the correlation. The LUS operators were blinded for the CT result, but not for the clinical picture. A 12-zone scanning technique was used to calculate the LUSS. The LUSS has been extensively used in ARDS and COVID-19 studies [6, 20]. See the Additional file 1: Digital Content and Additional file 2: Fig. S1 for the LUS scan protocol.

As there is no standardization of pleural line abnormalities and subpleural consolidation, their classification is thus subjective [21]. Ji et al. suggested a qualitative appreciation of the pleural line, where a normal pleural line scores: 0, an ‘irregular’ pleural line: 1, and a ‘blurred’ pleural line: 2 [10]. We tried to make a more reproducible, quantitative classification, with the following scores; a normal pleural line: 0; a thickened/irregular pleural line (without clear subpleural consolidations): 1; subpleural consolidations < 1 cm: 2; subpleural consolidations 1–2 cm: 3; subpleural consolidations 2–3 cm: 4; subpleural consolidations ≥ 3 cm (without progressing to tissue-like pattern): 5 [17]. The highest pleural abnormalities score was taken in each of the 12 zones.

### Statistical analysis

Baseline characteristics and outcome variables were presented as means ± standard deviations (± SD), medians and interquartile range (IQR), or numbers (percentages %) where appropriate. Groups were compared with independent Student’s *T* test, ANOVA, χ2-test, or Fisher’s exact test. A Shapiro–Wilk’s test, visual inspection of histograms, and Q–Q plots were used to determine data distribution. A two-sided significance level of 5% was used for all analyses, 95% confidence intervals (95%CI) are reported. All mixed models analyses were done with the maximum likelihood method. Statistical analyses were performed using SPSS IBM version 24.0 (SPSS Inc., Chicago, IL, USA).

#### Correlation

Given repeated measurements over time, linear mixed models analysis was used to estimate the relationship between the LUSS (± pleural abnormalities) and CTSS throughout the admission. We also assessed the relationship between the LUSS and CTSS on one hand, and the arterial oxygen partial pressure to fractional inspired oxygen (PaO2/FiO2) ratio, as well as the alveolar dead space to tidal volume ratio according to Enghoff (Vd/Vt ratio) on the other [22–24]. All estimated regression coefficients were standardized, so they can be interpreted as correlation coefficients. A coefficient of 0–0.19 indicated a slight; 0.20–0.39 a fair; 0.40–0.59 a moderate; 0.60–0.79 a substantial; and 0.80–1.0 an almost perfect correlation.

#### Agreement and measurement error between raters

We quantified reproducibility by assessing agreement and measurement error between raters for LUSS, pleural abnormalities, and CTSS by intra-class correlation coefficient (ICC) (two-way random model for agreement), and the smallest detectable change (SDC), respectively. The ICC is the degree of resemblance between sets of measurements expressed as a value between 0 and 1. The SDC represents the minimal change a score must show to ensure that the observed change is a true signal and not a product of measurement error (or imprecision) that may occur between raters. To visualize the SDC and limits of agreement (LoA) we also constructed Bland–Altman plots [25]. LUSS and pleural abnormalities were analyzed by AL and MHe, while the CTSS was analyzed by RvH and BT.

#### Mortality

We performed a linear mixed model analysis to assess if changes in pulmonary involvement graded by either LUSS (± pleural abnormalities) or CTSS are associated with mortality at different times during ICU admission. To do so, the development over time for pulmonary involvement was compared between survivors and deceased patients. Therefore, time (treated as a categorical variable represented by dummy variables), deceased (yes/no) and the interaction between time and deceased were added to the linear mixed model.

### Sample size

For the correlation between LUSS (± pleural abnormalities) and CTSS we consecutively included patients during the entire study period. However, taking into account a correlation of 0.67, alpha of 0.05 and beta of 0.20 at least 15 patients were needed [7].

For reproducibility between raters: both LUSS and CTSS previously described inter-rater agreement was excellent, albeit in the ED setting [1, 26]. The required sample size for both LUSS and CTSS was calculated based on an expected agreement of 0.88, a precision 0.1, and alpha of 0.05 and beta of 0.20. This resulted in a sample size of 21 patients. For the classification of pleural abnormalities and subpleural consolidations, there are no known agreement or measurement error parameters. We expected an agreement of 0.8, with a precision of 0.1, an alpha and beta of 0.05 and 0.20. This resulted in a required sample size of 51 for two raters. To avoid scan-location bias we included 60.

## Results

**Fig. 1:**
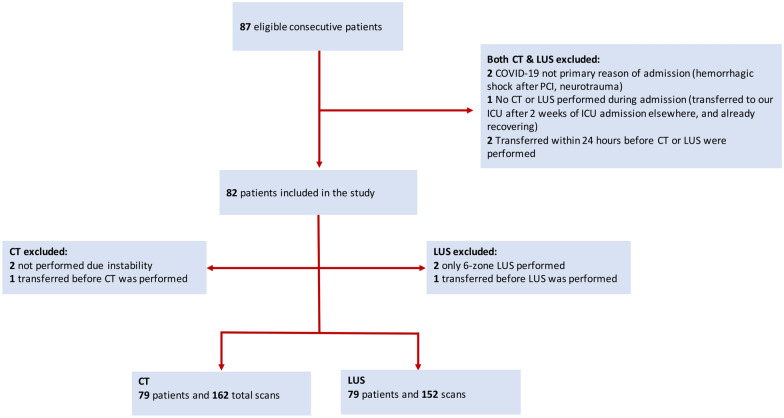
Flow of patient inclusion. CT: computed tomography. LUS: lung ultrasound

Of the 87 screened patients, 82 were included (Fig. 1). Patient characteristics are shown in Table 1. Compared to survivors, patients in the deceased group were older, had more comorbidities, higher creatinine, C-reactive protein (CRP), cardiac enzymes, initial positive end-expiratory pressure (PEEP) settings, and longer ICU admission time. Although the difference in CRP and PEEP between the two groups is not clinically relevant. A super-infection was suspected in 27.8% of CTs. In 74% of those cases either the right or the left lower lobe was involved, and in the remaining cases the posterior parts of the upper lobes were predominantly involved.

**Table 1:** Patient characteristics

	Total: 82	Alive: 50	Deceased: 32	*p* value
Demographics				
Age (years)	67 *[59–74]*	64 *[*5*7.8–70]*	71.5 *[65–76]*	**< 0.001**
Sex (male)	65 *(79.3)*	39 *(78)*	26 *(81.3)*	0.58
BMI (kg/m^2^)	29.5 (*6.1)*	30.1 *(5.5)*	28.9 *(6.6)*	0.47
APACHE II	12 *(10–13)*	12 (*10–12*)	12 (*10–13.3*)	0.38
SOFA score	7.5 *(2.9)*	7.2 *(2.9)*	8.1 *(2.9)*	0.17
Charlson Comorbidity Index	3.2 *(1.9)*	2.8 *(2.0)*	3.8 *(1.6)*	0.02
Laboratory parameters				
Creatinine (umol/L)	80.5 *[64.3–109.8]*	70 *[57.3–89.8]*	103.5 *[79.8–198.3]*	**< 0.001**
Leucocytes (× 10^9^/L)	10.2 *(4.9)*	9.8 *(4.6)*	10.8 *(5.5)*	0.43
CRP (mg/L)	139 *[90–190]*	128 *[83–183]*	159 *[105.5–193.3]*	**0.007**
Procalcitonin (ug/L)	0.35 *[0.18–1.06]*	0.27 *[0.13–0.62]*	0.75 *[0.20–1.48]*	0.42
LDH (U/L)	505 *[398–643]*	489 *[398–618]*	509 *[392–730]*	0.23
Hs Troponin T (ng/L)	24 *[12.5–51.5]*	18 *[9.8–38]*	39 *[19–119]*	**0.005**
NT-pro BNP (ng/L)	575 *[237–1011]*	472 *[193–749]*	756 *[395–1658]*	**< 0.001**
aPTT (sec)	26 [23–30]	25 [23–30]	26 *[23–29.5]*	0.96
d-Dimer (ng/mL)	1.65 *[1.04–5.74]*	1.42 *[0.94–5.11]*	2.96 *[1.18–6.38]*	0.87
Ventilation parameters				
PEEP (cm H2O)	11 *(3)*	11 *(2)*	12 *(3)*	**0.04**
PaO2/FiO2 ratio	105 *[75.8–147.5]*	111.4 *[75.8–147.5]*	105 *[75.6–154.6]*	0.96
etCO2 gap (kPa)	1.83 *(0.96)*	1.9 *(1.1)*	1.7 *(0.8)*	0.66
Initial imaging scores				
LUSS	21 *(5)*	*21 (5)*	*21 (6)*	0.55
Pleural abnormality score	13 *(7.6)*	*12 (6)*	*15 (9.5)*	0.14
CTSS	18 [16–22]	18 [16–21]	*19 [15–23.5]*	0.38
Outcomes				
ICU length of stay (days)	13 *[6–28.5]*	10 *[6–40.5]*	16 *[8.3–20]*	** < 0.001**
28-day mortality	29 *(35.4)*	N/A	N/A	
90-day mortality	32 *(39)*	N/A	N/A	

Values are n (%), mean (± SD), or median [IQR] as appropriate

*p* values comparing patients are from χ^2^-test, Fisher’s exact test, ANOVA or Mann–Whitney U test, with α = 0.05

APACHE II: Acute Physiology and chronic Health Evaluation II; aPTT: activated prothrombin time; BMI: Body Mass Index; CRP: C-reactive protein; EtCO2: end-tidal carbon dioxide; Hs: high sensitivity; IQR: inter-quartile range; ICU: intensive care unit; IU: international units; kPa: kilopascal; L: liter; LDH: lactate dehydrogenase; LUSS: lung ultrasound score; NT-pro BNP: N-terminal pro b-type natriuretic peptide; PE: pulmonary embolism; PEEP: positive end-expiratory pressure; PaO2/FiO2 ratio: ratio of arterial oxygen partial pressure to fractional inspired oxygen; Pleural abnormality score: separate pleural abnormality score, so without inclusion in the LUSS; SD: standard deviation; SOFA: Sequential Organ Failure Assessment

P-values in bold represent baseline characteristics that differ significantly

### Correlations

The correlation of CTSS with LUSS (without pleural abnormalities) was 0.45 (95% CI 0.31–0.59), while the correlation between CTSS with LUSS (with pleural abnormalities) was 0.31 (95% CI 0.15–0.46). CTSS was not correlated with the PaO2/FiO2 ratio, whereas the LUSS was significantly negatively correlated. The LUSS was fairly correlated with the Vd/Vt ratio, and the CTSS was only slightly correlated with the Vd/Vt ratio (Additional file 1: Table S1). Incorporation of pleural abnormalities to the LUSS did not lead to a significant improvement of the correlations.

### Agreement and measurement error between raters

**Fig. 2:**
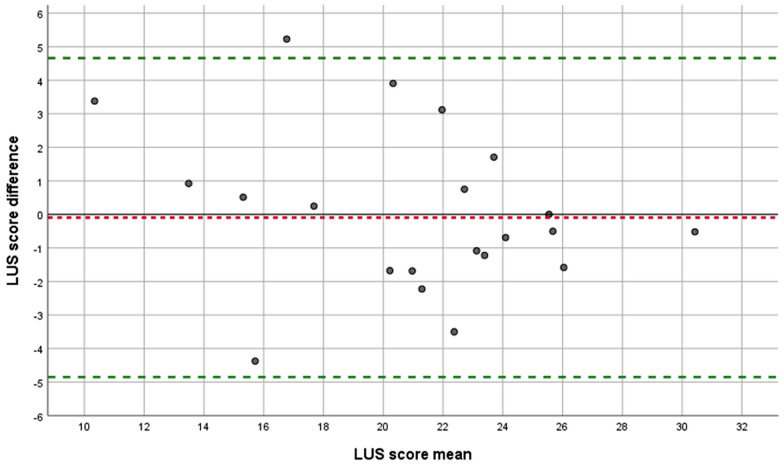
Bland–Altman plot for LUSS. LUSS: Lung ultrasound score. Each point represents agreement between the two raters AL and MHe. A jitter effect was added to improve visualization of data and avoid direct overlap of multiple examinations. Green dotted line: limits of agreement. Red dotted line: mean systematic difference

**Fig. 3:**
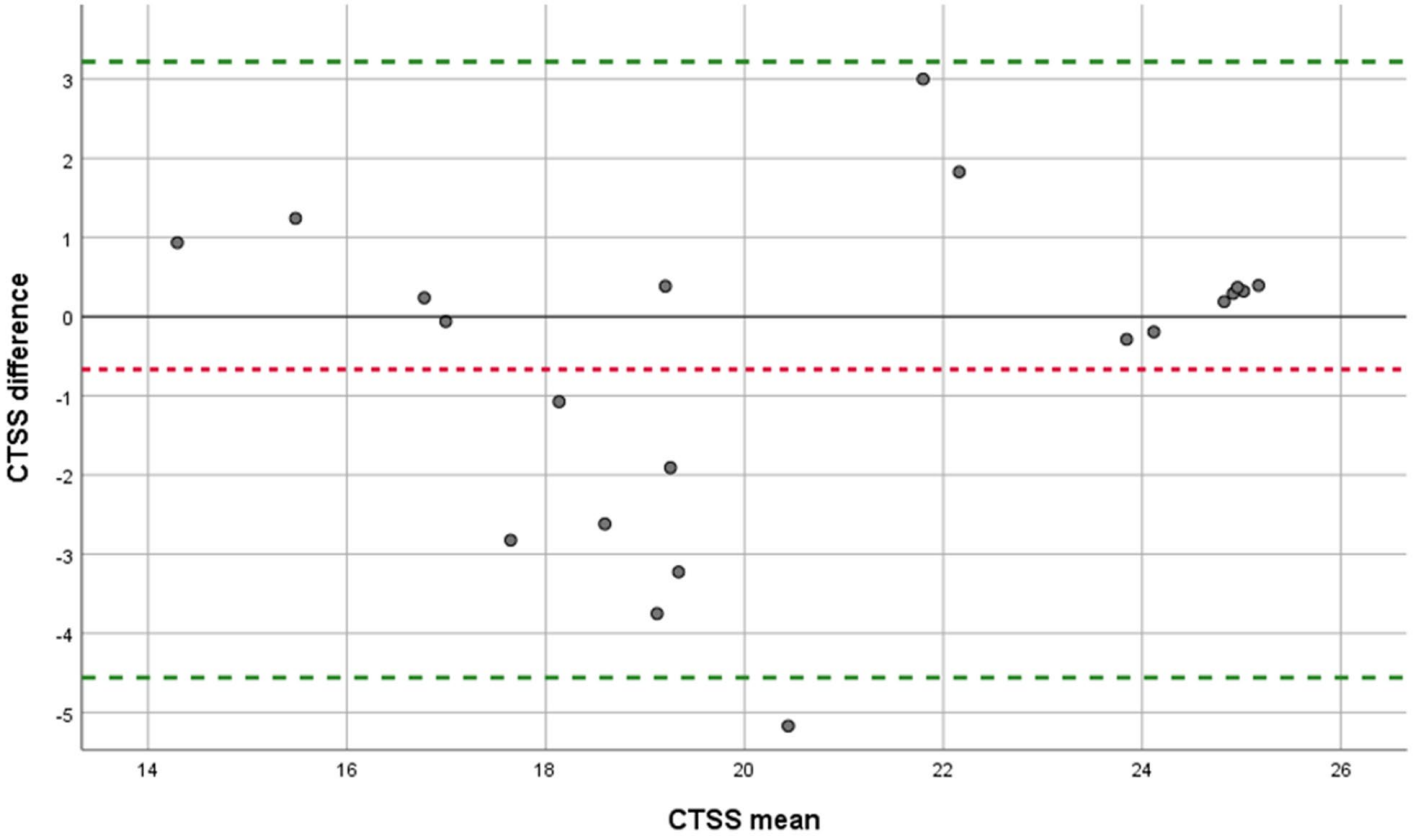
Bland–Altman plot for CTSS. CTSS: Computed tomography severity score. Each point represents agreement between the two raters RvH and BT. A jitter effect was added to improve visualization of data and avoid direct overlap of multiple examinations. Green dotted line: limits of agreement. Red dotted line: mean systematic difference

The ICC for LUSS was 0.88 (95% CI 0.73–0.95), while the SDC was 4.8. The ICC of pleural abnormalities was 0.94 (95% CI 0.90–0.96), while its SDC was 1.4. The ICC for CTSS was 0.84 (95% CI 0.65–0.93) and the SDC was 3.9. There was no proportional bias for LUSS or CTSS (Figs. 2 and 3). However, for pleural abnormalities the measurement error is less on the extremes of the spectrum: (0) ‘normal pleural line’ and (4, 5) ‘subpleural consolidation ≥ 2 cm’, compared to the categories in the middle [1] ‘thickened/irregular pleura’ to (3) ‘subpleural consolidations < 2 cm’ (Additional file 3: Fig. S2).

### Mortality

**Fig. 4:**
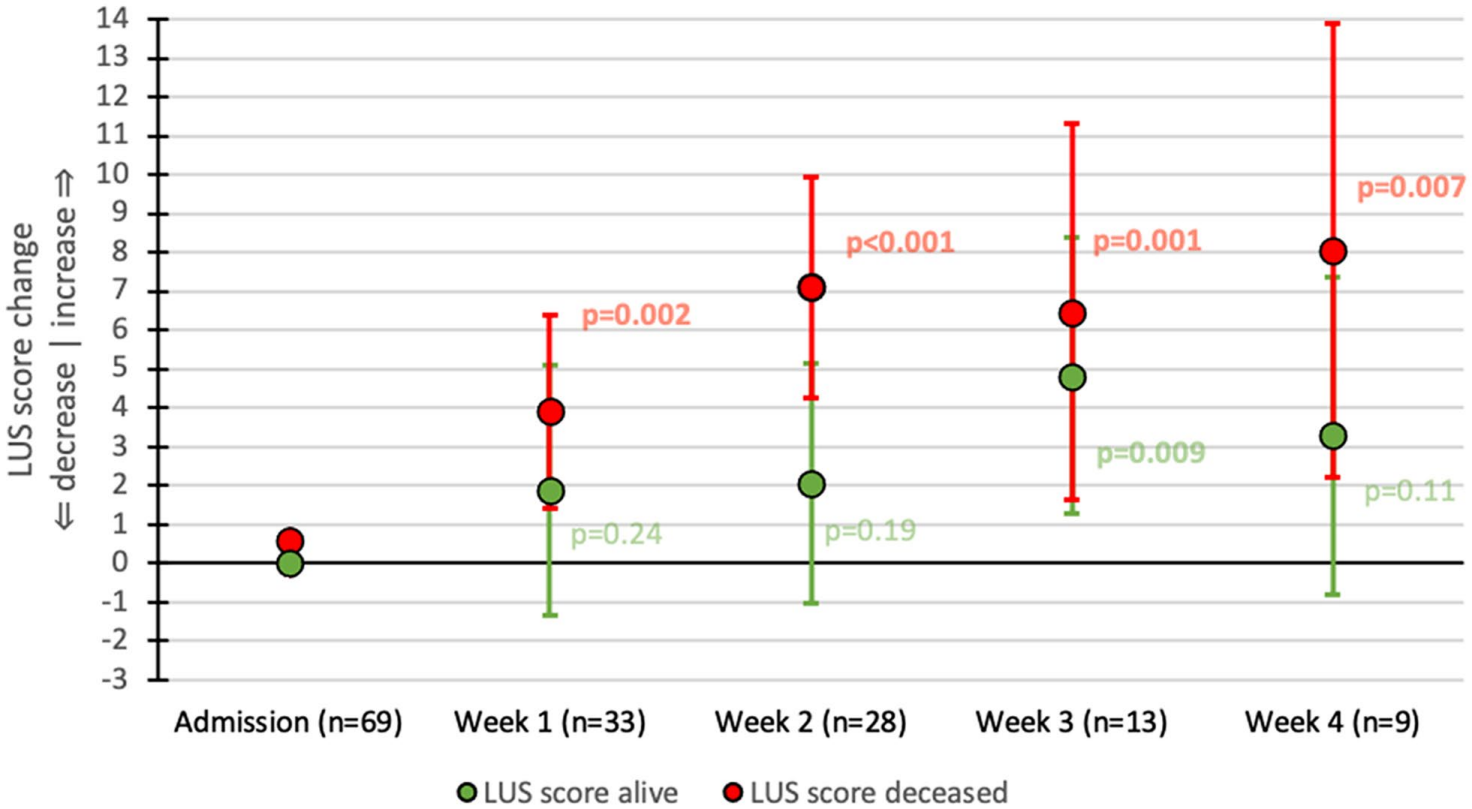
LUSS changes over time compared to admission. LUSS: lung ultrasound score. Each point represents the mean score change compared to respective admission with its 95% confidence interval. Green points: patients who survived. Red points: patients who died. *P* values at follow-up points should be interpreted as the significance of the change of that particular point compared to baseline. Significant *p* values are in bold. At admission, there was no difference between the LUSS of patients who survived until discharge or died during admission (*p* = 0.67)

In deceased patients a significant rise in LUSS was found compared to admission (Fig. 4). For survivors the difference compared to admission only became significant in week 3 for LUSS. However, the difference between survivors and deceased patients for LUSS was only significant after 2 weeks of admission; 5.62 (95% CI 2.44–3.99), which became insignificant afterward (Additional file 4: Fig. S3). The addition of pleural abnormalities did not improve the association with mortality (Additional file 5: Fig. S4).

**Fig. 5:**
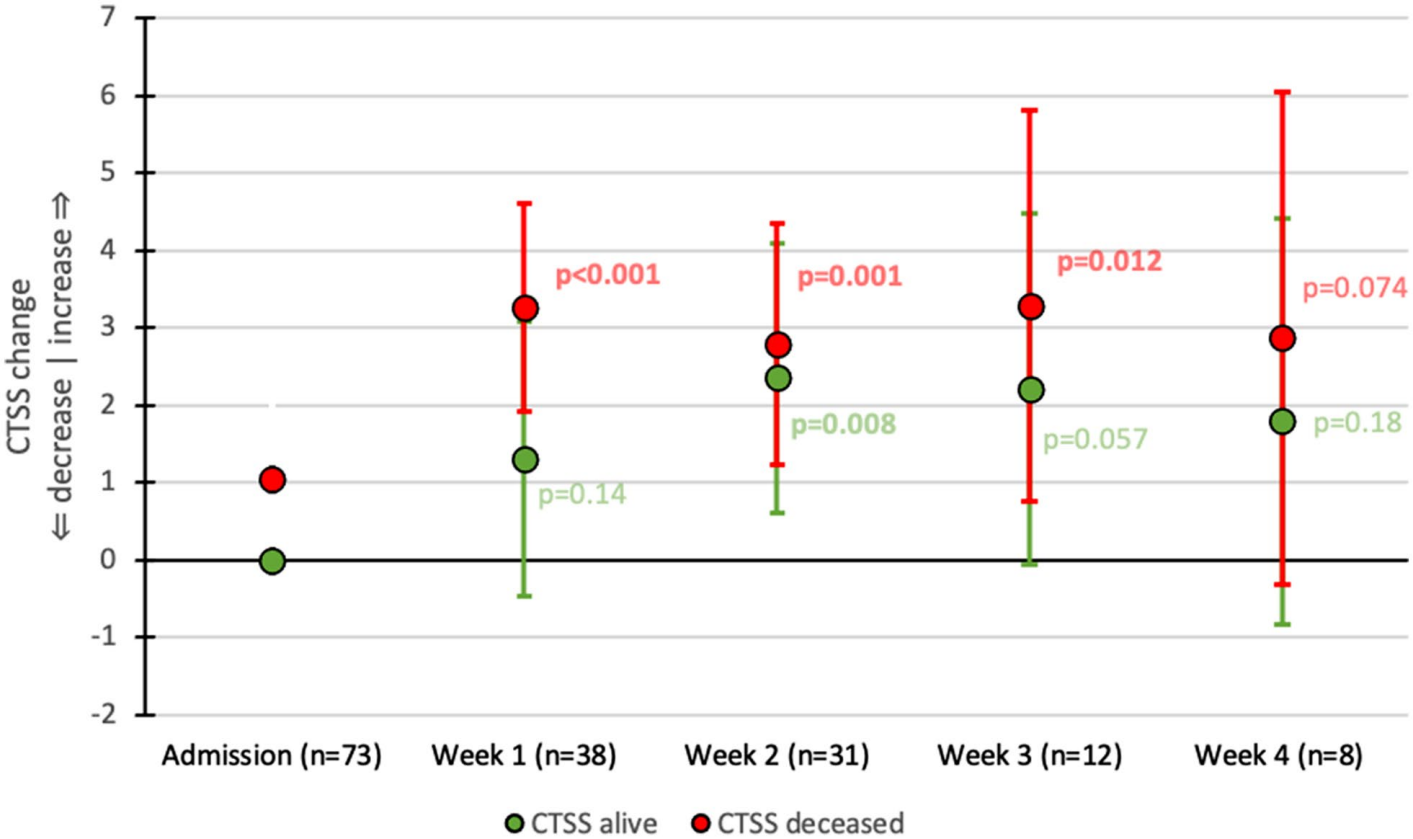
CTSS changes over time compared to admission. CTSS: Computed tomography severity score. Each point represents the mean score change compared to respective admission with its 95% confidence interval. Green points: patients who survived. Red points: patients who died. *P* values at follow-up points should be interpreted as the significance of the change of that particular point compared to baseline. Significant *p* values are in bold. At admission, there was no difference between the CTSS of patients who survived until discharge or died during admission (*p* = 0.26)

In deceased patients CTSS rose significantly compared to baseline in the first week of admission, and remained stable afterward (Fig. 5). In survivors there was a slower and smaller upward trend in CTSS until week 2 which also stabilized afterward. The difference in CTSS between deceased patients and survivors was only significant after week 1; 3.0 (95% CI 0.38–5.62), but not afterward (Additional file 6: Fig. S5).

## Discussion

The main findings of this prospective, observational study assessing the ability LUS and CT to monitor pulmonary involvement in COVID-19 ICU patients are:

(1) LUSS has a moderate correlation with the CTSS for the quantification of pulmonary involvement, and a better correlation with the PaO2/FiO2 and Vd/Vt ratio than CTSS; (2) Serial LUSS is capable of detecting true changes in pulmonary involvement, whereas CTSS cannot; (3) Of the two scores, only a rise in LUSS after 2 weeks is significantly associated with mortality and detectable beyond measurement error. However, this association becomes insignificant again in the weeks afterward; (4) Addition of subpleural abnormalities did not lead to improvement of the existing LUSS.

Our results suggest LUS might be used as a substitute of chest CT for the monitoring of COVID-19 pneumonia severity in COVID-19 ICU patients. The correlation of the total LUSS and CTSS was moderate, which is comparable to previous studies [6, 7, 16]. Surprisingly, the CTSS did not correlate with PaO2/FiO2 ratio, and only correlated slightly with the Vd/Vt ratio. Even though the measurements were taken at time of CT. The LUSS correlated fairly with both. A worsening of the PaO2/FiO2 ratio over time would prompt a repeat CT in clinical practice to evaluate the progression of pulmonary involvement, but this thus seems redundant.

Although the degree of pulmonary involvement at presentation to the emergency department is associated with ICU admission and adverse events [6–10], previous studies failed to show an association between LUSS or CTSS at ICU admission and mortality (without it being incorporated in a composite end-point) [1, 7, 8, 10, 16, 27]. The only other prospective study assessing the prognostic value of serial LUS and CT, showed that the LUSS after 1 week did not differ significantly from the LUSS at admission, and did not show an association with mortality. However, our study showed that the LUSS might help differentiate between patients who survive or die if you follow them longer. Although the LUSS did not exceed the SDC (4.8) on a week-to-week basis, from week 2 onward the LUSS exceeded the SDC, and was significantly higher compared to admission in deceased patients. The mean difference between deceased patients and survivors was significant and also exceeded the SDC in week 2. This was not the case in weeks 3 and 4, possibly owing to the small sample size, as results did show a trend. In patients who survived, both LUSS and CTSS showed no decrease over time, stressing the discrepancy between clinical recovery and lack of image resolution [16].

Contrary to the LUSS, the CTSS seems not useful in monitoring ICU COVID-19 patients, which is at odds with the claims of the original CO-RADS protocol, which promoted the CTSS as a tool for follow-up [28]. Even though there was a statistically significant difference between patients who survived and those who died in week 1, it falls within the measurement error. Indeed, the CTSS never changed more than its SDC (3.9); neither between admission and following weeks, nor between surviving and deceased patients. This signifies that measurement errors based on interpretative differences between CTSS raters are greater than the true variation in score (based on actual pulmonary involvement changes). Thus, in clinical practice, the CTSS measurement error may suppress the true CTSS signal, i.e., a clinical CTSS change is more likely to be caused by measurement error than disease progression. This may be explained by the fact that the CTSS uses categories that are too broad, lacks responsiveness, and suffers from ceiling effects [25, 29]. In addition, the CTSS only includes ground glass opacities, omitting consolidations and fibrosis which are present later in the disease [13–16, 28]. Their incorporation might improve the CTSS. Other suggestions are increasing the number of categories of the CTSS, or using use of artificial intelligence (AI) to aid pulmonary involvement quantification [30].

Although there are other reasons to perform a CT—i.e., suspicion of a pulmonary embolism, or a super-infection—it could be argued that even for these indications CT does not have to be the first diagnostic step and the amount of CTs could at least be reduced. For instance, less invasive methods like routine screening of deep venous thrombosis by POCUS could obviate the need for further a CT pulmonary angiography [31–33]. Furthermore, although there is some evidence in immunocompromised patients that CT-guided broncho-alveolar lavage (BAL) results in a higher yield [34], BAL can also be performed without CT guidance [35]. In our cohort, a super-infection was suspected in 27.8% of CTs, which is comparable to what is reported in the most recent meta-analysis [36]. Since we found the posterior parts of the lungs were primarily involved when superinfection was suspected, we argue that a CT is not required to determine the optimal BAL location and these areas could be sampled empirically.

Some of the criticisms of the CTSS also apply to the LUSS, particularly the fact that its categories are too broad. However, we showed that including pleural abnormalities unfortunately did not improve the LUSS to a significant degree. First, this might be due to the fact that the LUSS already incorporates pleural abnormalities and consolidations to some degree, so by adding them again you might be counting them twice [20]. Second, we found there is more measurement error in pleural abnormality categories 1–3, than categories 0 and 4–5. This is no surprise as there is no clear definition of a ‘thickened’, ‘blurred’, or ‘irregular’ pleural line, nor is it clear when an ‘irregular’ pleura is actually caused by clear subpleural consolidation. As such clinicians will interpret these constructs in different ways [10, 17]. Moreover, it is unclear what the clinical relevance of pleural abnormalities of varying degrees is. As our results show they might not hold any additional value. In any case, universal standardization of these signs—as was done with other LUS signs—is paramount to facilitate comparison across studies [21]. All in all, we argue to keep using the existing LUSS—which has already been validated in multiple settings—and that restraint should be exercised in creating new, more complex and/or unvalidated measurement instruments [20, 25].

Maybe in the future AI can help improve LUS quantification of pulmonary involvement, and elucidate which findings have additional prognostic value. It has shown promise in a few studies, but further innovation and research is required before it will be ready for use on a larger scale [20, 37–39]. An important condition for AI-based quantification is that it should be directly applicable and useable at the bedside, so the benefits of the point-of-care nature of LUS are not lost.

In summary, we argue that if one would like to monitor ICU COVID-19 patients with an imaging modality, LUSS should be preferred over CTSS, at least as an initial step. Especially considering the cost, safety and time disadvantages associated with CT and the lack of its availability in many parts of the world.

### Strengths and limitations

First, this was a single center study with a relatively limited sample size. Still, this is the largest prospective study in consecutive COVID-19 ICU patients to investigate the ability of serial LUS and CT in monitoring and prognostication. Second, although we are a tertiary center, our case mix was reflective of the total ICU population, since ICU patients were divided across the country according to a fair-share principle. Accordingly, we believe selection bias was, therefore, minimized. Third, time between LUS and CT was always below 24 h, which limited imprecision of the correlations. Fourth, we only included patients who deteriorated or stagnated in their recovery, and did not perform a CT or LUS in patients that were recovering nor in patients in which the disease progressed to such a degree that further diagnostics and treatment were deemed no longer useful. Although, this does reflect daily practice, it would be interesting to include these groups in further research as well. Fourth, operators were blinded for CT results, reducing information bias to a minimum.

## Conclusions

LUSS correlated with CTSS throughout ICU admission but performed similar or better at agreement between raters and mortality prognostication. Incorporation of pleural abnormalities did not improve the LUSS. Given the benefits of LUS over CT, LUS should be preferred over CT as the initial monitoring tool.

## Supplementary Information

**Additional file 1:** Supplementary Material including our ICU treatment protocol, the CT scan protocol, LUS scan protocol. **Table S1** Correlations of different scores of pulmonary involvement with respiratory parameters.

**Additional file 2: Figure S1.** Lung ultrasound scan zones (only right side shown).

**Additional file 3: Figure S2.** Bland–Altman plot for pleural abnormalities on LUS.

**Additional file 4: Figure S3.** Difference in LUSS: alive versus dead patients.

**Additional file 5: Figure S4.** Difference in LUSS + pleural abnormalities: alive versus dead patients.

**Additional file 6: Figure S5.** Difference in CTSS: alive versus dead patients.

## Data Availability

The data sets used and/or analyzed during the current study are available from the corresponding author on reasonable request.

## Abbreviations

AI: Artificial intelligence
BAL: Bronchoalveolar lavage
CI: Confidence interval
Cm: Centimeter
CO-RADS: COVID-19 Reporting and Data System
COVID-19: Coronavirus Disease 2019
CRP: C-reactive protein
CT: Computed tomography
CTSS: CT severity score
ICC: Intra-class correlation coefficient
ICU: Intensive care unit
IQR: Interquartile range
LUS: Lung ultrasound
LUSS: Lung ultrasound score
PaO2/FiO2 ratio: Arterial oxygen partial pressure to fractional inspired oxygen ratio
PEEP: Positive end expiratory pressure
POCUS: Point-of-care ultrasound
SD: Standard deviation
SDC: Smallest detectable change
Vd/Vt ratio: Alveolar dead space to tidal volume ratio
