# Supplementary material for: Monitoring of pulmonary involvement in critically ill COVID-19 patients - should lung ultrasound be preferred over CT?
Source: Ultrasound J. 2023 Feb 26;15:11. doi: 10.1186/s13089-022-00299-x (PMC9968403; doi:10.1186/s13089-022-00299-x)
Supplement: Supplementary file 1 — Additional file 1: Supplementary Material including our ICU treatment protocol, the CT scan protocol, LUS scan protocol. Table S1 Correlations of different scores of pulmonary involvement with respiratory parameters. [file 13089_2022_299_MOESM1_ESM.docx]

# Supplementary Material

## The ICU-treatment protocol

Patients admitted to our ICU were treated with selective intestinal decontamination (Tobramycin 80mg/Colisitin 100mg/Nystatin 2.000.000 IU) 10ml suspension orally or via nasogastric tube(s) thrice daily, and 0.5mg mouthpaste thrice daily for the duration mechanical ventilation. In addition, we prescribed intravenous Ceftriaxon 2 grams once daily for four days. We also treated patients with intravenous Dexamethasone 6 grams once daily for 7 days. IL-6 inhibitors (e.g., Tocilizumab or Sarilumab) were not used yet during this study. Given the high incidence of the venous thromboembolism reported in the literature from the first wave, the protocol in our ICU was to increase the dosing interval of prophylactic low molecular weight heparin (Fraxiparin). Patients <100 kg received 2850IE bidaily instead of once daily, while patients >100kg received 5700IE bidaily instead of once daily. From the third dose onwards, anti-factor Xa (anti-fXa) measurements were performed daily. The anti-fXA target level was 0.1-0.5 U/ml. When levels remained <0.1 U/ml, physicians could decide to increase the dose to get it within target range. When patients required therapeutic anticoagulation intravenous unfractionated heparin (UFH) was used, with a target activated partial thromboplastin time (aPTT) of 45-60. When no therapeutic aPTT levels of UFH were reached despite high doses (>25IE/kg/hour), anti-fXa levels could be used to guide therapy, with a therapeutic target range of 0.3-0.7 IE/ml. All mechanically ventilated patients were treated with pressure control or pressure support, adhering to lung protective protocols advised by the Dutch Society of Intensive Care (NVIC) (<https://nvic.nl/covid-19>: Heunks et al. 2020).

## The CT scan protocol

Chest CT was performed with two multidetector CT scanners: Siemens Somatom Drive (Siemens Healthineers, Erlangen, Germany), and a GE Discovery 750 HD (GE Healthcare, Milwaukee, MI). All patients underwent CT scanning of the chest during end-inspiration. Slice thickness for all scanners was between 0.625-1.25 mm.

## The LUS scan protocol

All examinations, were performed or supervised by ICU residents certified in ultrasound using a COVID-19 unit-restricted SonoSite-Edge II ultrasound machine. A 10-5 MHz linear transducer or 5-3 MHz curvilinear transducer with lung examination setting was used. The lung ultrasound score (LUSS) is calculated in six zones per hemithorax: anterior, lateral, and posterior; split by sternum, anterior, and posterior axillary lines respectively, and divided into superior and inferior regions (Supplementary Figure 1). The LUSS has the following four progressive categories: 0, normal aeration (A-lines or ≤2 B-lines); 1, moderate loss of aeration (three or more well-spaced B-lines); score 2, severe loss of aeration (B-lines or subpleural consolidations in ≥50% of the zone); and score 3, complete loss of aeration (tissue-like pattern). The total LUSS sums the scores from all 12 zones, creating a final score range from 0 (all regions are well aerated) to 36 (all regions are consolidated).

# Additional Tables

### Table S1 – Correlations of different scores of pulmonary involvement with respiratory parameters

|  | LUSS | LUSS + pleural abnormalities | CTSS |
| --- | --- | --- | --- |
| PaO2/FiO2 ratio | -0.31  *(-0.48 - -0.16)* | -0.20  *(-0.37 - -0.04)* | -0.11  *(-0.26 - 0.05)* |
| Vd/Vt | 0.20  *(0.02 - 0.38)* | 0.25  *(0.07 – 0.43)* | 0.18 *(0.003 - 0.35)* |

All values are given with their 95% confidence interval in italic. CTSS: computed tomography severity score; LUSS: lung ultrasound score; PaO2/FiO2 ratio: arterial oxygen partial pressure to fractional inspired oxygen ratio; Vd/Vt ratio: the alveolar dead space to _tidal_ volume ratio.

# Additional Figure Legends

## Figure S1 – Lung ultrasound scan zones (only right side shown)

## Figure S2 – Bland-Altman plot for pleural abnormalities on LUS

LUS: lung ultrasound. Each point represents agreement between the two raters AL & MHe. A jitter effect was added to improve visualization of data and avoid direct overlap of multiple examinations. Green dotted line: limits of agreement. Red dotted line: mean systematic difference.
A normal pleural line: 0; a thickened/irregular pleural line (without clear subpleural consolidations): 1; subpleural consolidations <1cm: 2; subpleural consolidations 1-2cm: 3; subpleural consolidations 2-3cm: 4; subpleural consolidations ≥3cm (without progressing to tissue-like pattern): 5.

## Figure S3 – Difference in LUSS: alive versus dead patients

LUSS: lung ultrasound score. Each blue point represents the mean score difference between alive and dead patients per week of admission with their 95% confidence interval. P-values should be interpreted as the significance of the change of that particular point compared to baseline. Significant p-values are in bold.

## Figure S4 – Difference in LUSS + pleural abnormalities: alive versus dead patients

LUSS: lung ultrasound score. Each blue point represents the mean score difference between alive and dead patients per week of admission with their 95% confidence interval. P-values should be interpreted as the significance of the change of that particular point compared to baseline. Significant p-values are in bold.

## Figure S5 – Difference in CTSS: alive versus dead patients

CTSS: Computed tomography severity score. Each blue point represents the mean score difference between alive and dead patients per week of admission with their 95% confidence interval. P-values should be interpreted as the significance of the change of that particular point compared to baseline. Significant p-values are in bold.
