# Supplementary figures and images for: Monitoring of pulmonary involvement in critically ill COVID-19 patients - should lung ultrasound be preferred over CT?
Source: Ultrasound J. 2023 Feb 26;15:11. doi: 10.1186/s13089-022-00299-x (PMC9968403; doi:10.1186/s13089-022-00299-x)

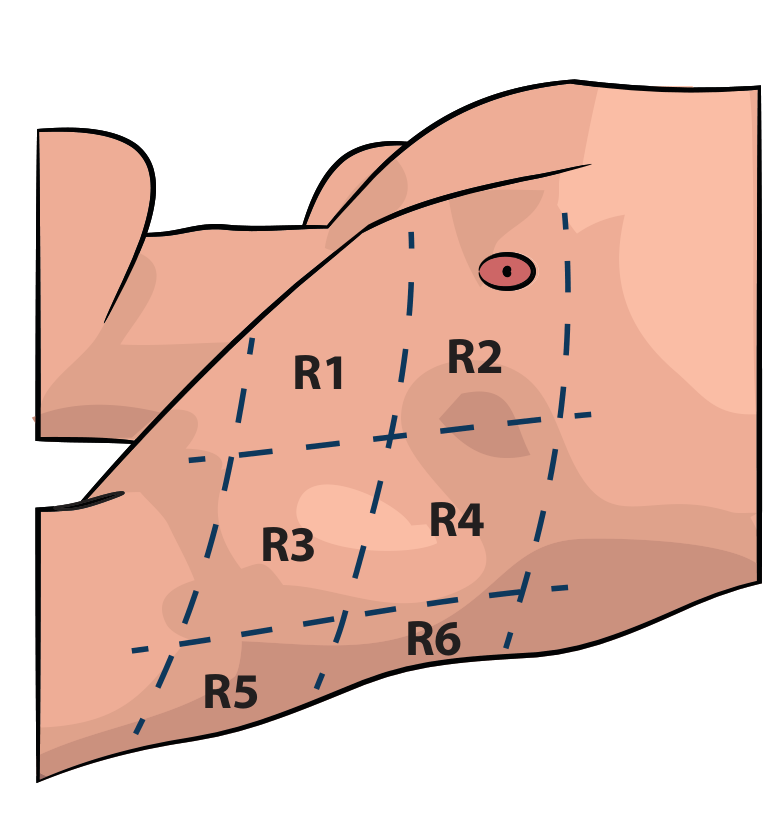

Supplement: Supplementary file 2 — Additional file 2: Figure S1. Lung ultrasound scan zones (only right side shown). [file 13089_2022_299_MOESM2_ESM.tiff]

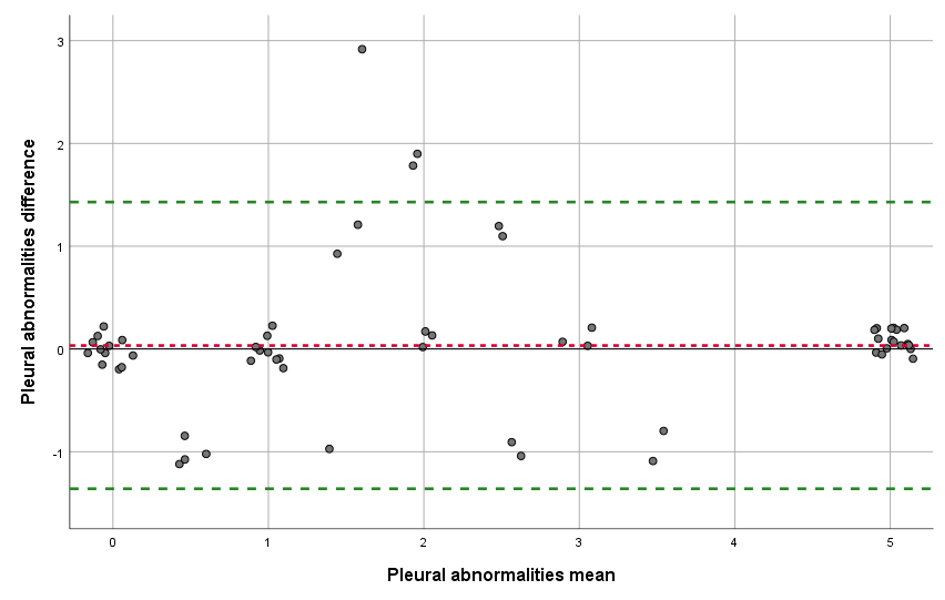

Supplement: Supplementary file 3 — Additional file 3: Figure S2. Bland–Altman plot for pleural abnormalities on LUS. [file 13089_2022_299_MOESM3_ESM.tiff]

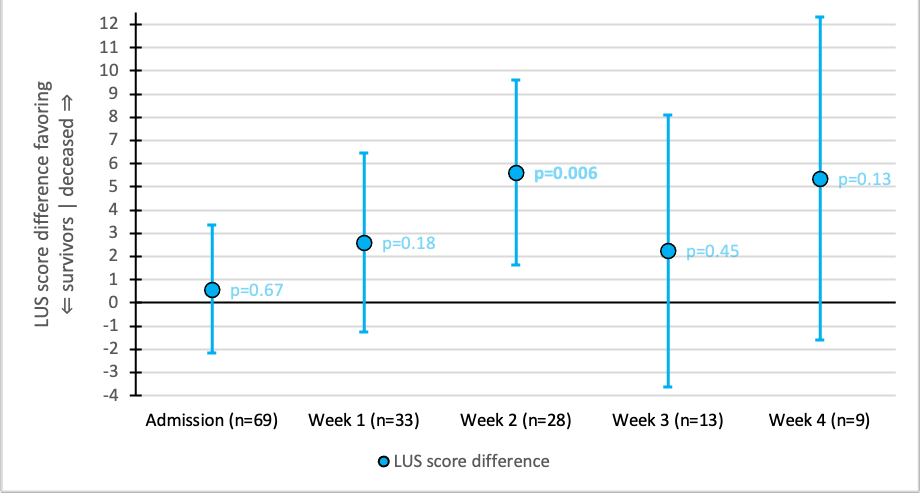

Supplement: Supplementary file 4 — Additional file 4: Figure S3. Difference in LUSS: alive versus dead patients. [file 13089_2022_299_MOESM4_ESM.tiff]

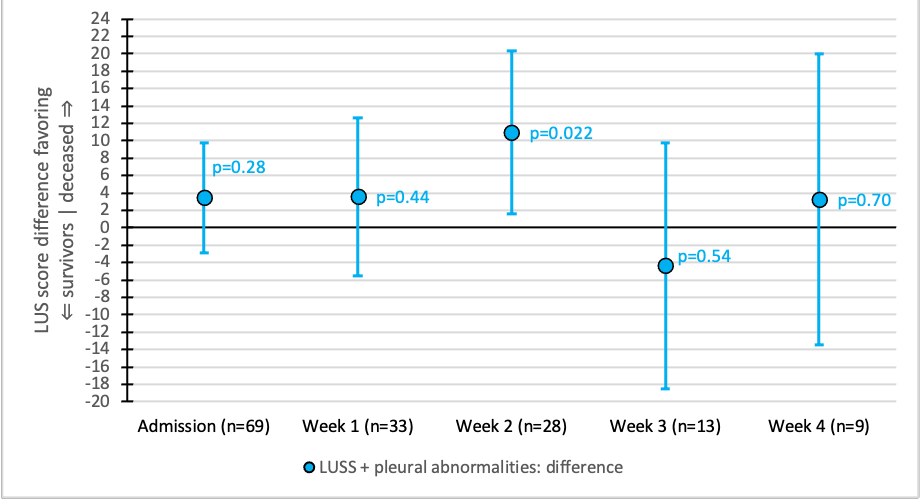

Supplement: Supplementary file 5 — Additional file 5: Figure S4. Difference in LUSS + pleural abnormalities: alive versus dead patients. [file 13089_2022_299_MOESM5_ESM.tiff]

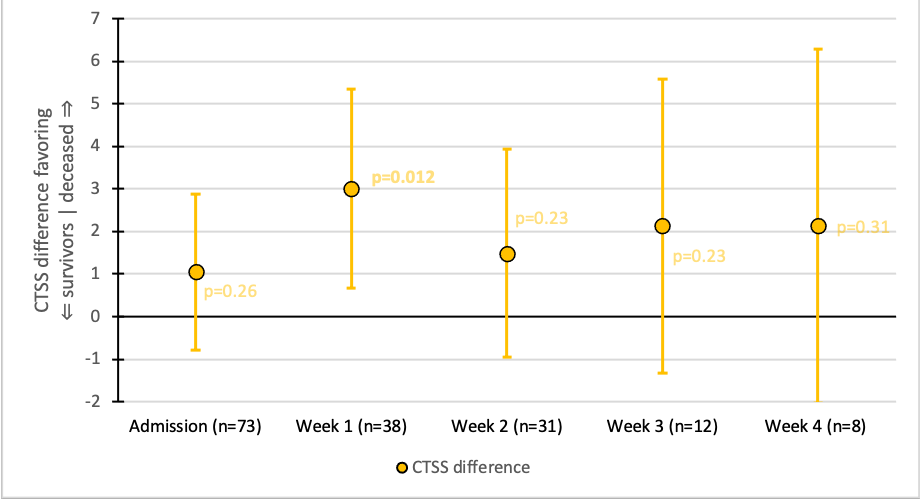

Supplement: Supplementary file 6 — Additional file 6: Figure S5. Difference in CTSS: alive versus dead patients. [file 13089_2022_299_MOESM6_ESM.tiff]
